# The Prevalence of Integument Injuries and Associated Risk Factors Among Canadian Turkeys

**DOI:** 10.3389/fvets.2021.757776

**Published:** 2022-01-07

**Authors:** Emily M. Leishman, Nienke van Staaveren, Vern R. Osborne, Benjamin J. Wood, Christine F. Baes, Alexandra Harlander-Matauschek

**Affiliations:** ^1^Department of Animal Biosciences, Centre for the Genetic Improvement of Livestock, University of Guelph, Guelph, ON, Canada; ^2^Department of Animal Biosciences, The Campbell Centre for the Study of Animal Welfare, University of Guelph, Guelph, ON, Canada; ^3^Department of Animal Biosciences, The Centre for Nutrition Modelling, University of Guelph, Guelph, ON, Canada; ^4^Hybrid Turkeys, Kitchener, ON, Canada; ^5^School of Veterinary Science, University of Queensland, Gatton, QLD, Australia; ^6^Vetsuisse Faculty, Institute of Genetics, University of Bern, Bern, Switzerland

**Keywords:** animal welfare, injurious pecking damage, management, Meleagris gallopavo, stockmanship, survey

## Abstract

Injurious pecking can cause a wide range of damage and is an important welfare and economic issue in turkey production. Aggressive pecking typically targets the head/neck (HN) area, and feather pecking typically targets the back/tail (BT) area; injuries in these separate areas could be used as a proxy for the level of aggressive and feather pecking in a flock. The objective of this study was to identify risk factors for integument injuries in Canadian turkey flocks. A survey containing a questionnaire about housing and management practices and a scoring guide was distributed to 500 turkey farmers across Canada. The farmer scored pecking injuries in two different body areas (HN and BT) on a 0–2 scale on a subset of birds within each flock. Multivariable logistic regression modeling was used to identify factors associated with the presence of HN and BT injuries. The prevalence of birds with integument injuries ranged widely between the flock subsets (HN = 0–40%, BT = 0–97%), however the mean prevalence was low (HN = 6%, BT = 10%). The presence of injuries for logistic regression was defined as flocks with an injury prevalence greater than the median level of injury prevalence in the dataset (3.3% HN and 6.6% BT). The final logistic regression model for HN injuries contained five variables: flock sex, flock age, number of daily inspections, number of different people during inspections, and picking up birds during inspections (N = 62, pR^2^ = 0.23, α = 0.05). The final logistic regression model for BT injuries contained six variables: flock sex, flock age, litter depth, litter condition, inspection duration, and use of hospital pens for sick/injured birds (N = 59, pR^2^ = 0.29, α = 0.05). Flock age, and to a lesser extent, sex was associated with both types of injuries. From a management perspective, aggressive pecking injuries appear to be influenced by variables related to human interaction, namely during inspections. On the other hand, the presence of feather pecking injuries, was associated with litter condition and other management factors like separating sick birds. Future research on injurious pecking in turkeys should focus on these aspects of housing and management to better describe the relationship between the identified variables and the prevalence and severity of these conditions.

## Introduction

Both wild and domestic birds peck as part of their natural behavior for many reasons. For example, they use their beaks to feed, communicate, or fight for control or dominance (1). In domestic birds kept in free-range and conventional housing systems, such as laying hens, broiler breeders, ducks, and turkeys, injurious pecking is often reported (1). Injurious pecking is an umbrella term for a problematic group of bird-to-bird behaviors in poultry flocks, including turkeys (1, 2). This term is named after the consequences (injuries) to the feathers, skin, or outgrowths (1).

First described by Savory (3), injurious pecking can be broken down into three main types of pecking: aggressive pecking, tissue pecking (or cannibalistic pecking), and severe feather pecking. Aggressive pecking targets the head, neck, and snood areas in turkeys (3–5) and tends to be related to establishing/maintaining social hierarchy (6, 7), although it can escalate into violence with no apparent function (5). Severe feather pecking is suggested to be a redirected foraging behavior, possibly from frustration due to a lack of environmental enrichment [“ethological view”: (1, 7) or, alternatively, a result of changes in brain structure similar to neuropsychiatric disorders [“dysfunctional view”: (1, 8)]. Typically, this behavior targets the wings, back, and/or tail, and pecking bouts can cause substantial feather loss (2, 3). These denuded areas can then become targets for tissue pecking, which can result in tissue damage and bleeding (1). Overall, injurious pecking can cause injuries and feather loss to certain body areas, which can be scored as indicators of aggressive and severe feather pecking behaviors (9).

Injurious pecking has been identified as a key welfare problem in turkeys (10) and a source of economic losses (11). Duggan et al. (12) reported that 58% of dead or culled birds in eight studied flocks (~50,000 birds) had evidence of severe pecking injuries. Cannibalism has been reported as one of the leading causes of on-farm mortality by Canadian turkey farmers, and skin conditions were reported as one of the main reasons for condemnations at the processing plant (13). If feathers are pecked (dead structure), it does not cause harm *per se*, but it impairs the structure/function of the feathers (1). Feathers are necessary for thermoregulation. Birds that display damage to their feather cover lose more heat and increase their feed intake to increase their metabolic heat production, resulting in yet another source of economic loss in the form of reduced feed efficiency (11).

The exact etiology of injurious pecking in domestic turkeys is unknown. It is generally described as a multifactorial problem that makes prevention and control challenging, especially considering the involvement of genetic and management components (1, 2). Kanis et al. (14) identified two promising strategies for improving farm animal welfare: selective breeding for desired traits and improving housing and management conditions. While farmers tend to have little control of genetic breeding programs and decisions, they can make changes to their housing and management practices more easily. Injurious pecking in turkeys is known to be influenced by a variety of environmental parameters such as housing system (12), floor space (15), group size (16), or lighting (17, 18). For example, turkeys in natural sided (curtain) barn environments exhibit more aggressive pecking than flocks housed in fully enclosed barns, possibly due to more frequent or substantial fluctuations in lighting, temperature, and humidity (12). Aggressive interactions have also been more frequent with less floor space (15) and smaller group sizes (16). Other management decisions like lighting type (i.e., incandescent, fluorescent), may distort turkeys' perception of ultraviolet markings on the feathers of their flock mates and make these areas targets for severe feather pecking (17–20).

Several studies have investigated injurious pecking behavior in North American turkey flocks (4, 12, 21). However, there is a lack of risk factor assessments to identify variables associated with pecking injuries in turkeys in Canada. To improve housing and management conditions and improve turkey welfare, we first need to determine which conditions influence the prevalence of relevant traits or behaviors. Since it is not always feasible to conduct behavioral assessments of pecking behavior on a large scale, looking at the aftermath of the behavior (injuries) can be used as a proxy for the level of injurious pecking in a flock. Therefore, the objective of this study was to investigate the factors influencing the prevalence of injurious pecking-related injuries in domestic turkey flocks in Canada.

## Materials and Methods

### Questionnaire

A two-part survey was disseminated to turkey farmers in Canada to identify factors associated with the prevalence of pecking injuries. The first component included a questionnaire comprised of mainly closed questions about a variety of production aspects such as bird characteristics (i.e., sex, age, weight), housing (i.e., lighting, air quality), management (i.e., litter, feed, water), and health (i.e., vaccinations, diseases, biosecurity). The questionnaire topics can be found in Supplementary Material 1 and a descriptive analysis of these data is included in van Staaveren et al. (22). Pilot testing of the questionnaire was conducted with collaborators in the turkey industry (farmers, veterinarians, genetic and feed company representatives) to test completion time and verify that the questions were meaningful for the Canadian turkey production sector and easy for farmers to understand. The final questionnaire was estimated to take 1.0–1.5 h to complete.

### Scoring Guide for Pecking Injuries

The second component of the survey was a scoring guide for pecking injuries. Since injuries to the head/neck (**HN**) area and back/tail (**BT**) area can reflect different behavioral motivations (aggressive and feather pecking, respectively), farmers were asked to score these areas separately (23). In this case, injuries are defined as damage to the skin as well as feather damage or denuded areas; in other words, any disturbance to the structure or function of a body area (1).

**Figure 1:**
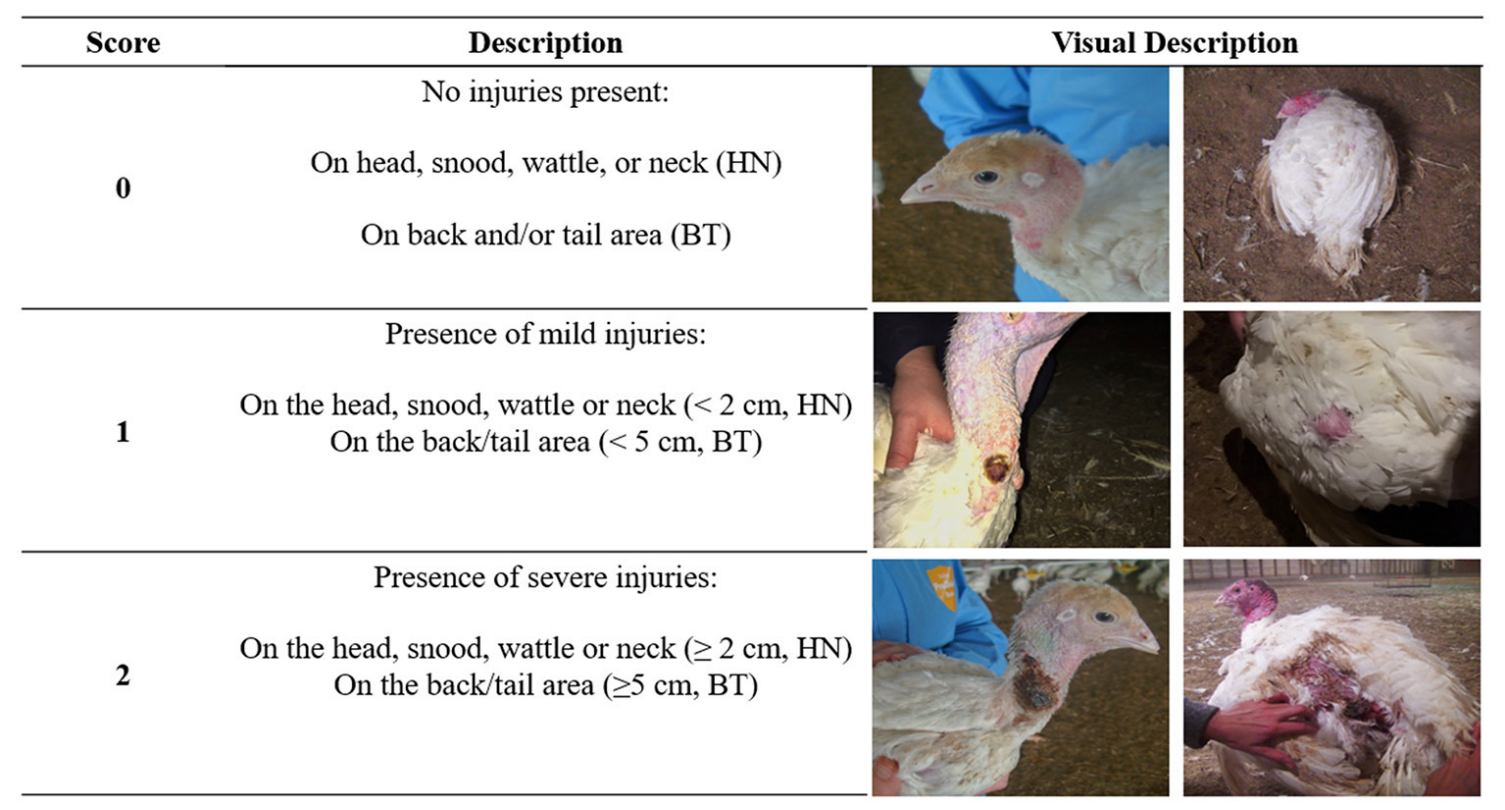
Simplified scoring system for farmers to score head/neck (HN, left images) and back/tail (BT, right images) on a subset of turkeys in their flock.

Detailed visual and written instructions were provided to describe the scoring methodology, including the types of injuries, selection of birds, and recording of data. Injuries to the HN and BT areas were scored on a three-point scale according to severity (Figure 1, adapted from Knierim et al., 24) by each farmer on a subset of 30 birds in their flock spread throughout the barn. The sample size of 30 birds was chosen after discussion with industry stakeholders and it was determined that any larger number could not be completed within the scope of a normal daily inspection and would therefore result in a substantially lower response rate. Instructions were provided within the scoring guide for choosing a proportional sample of birds from different areas of the barn. In particular, farmers were asked to score 10 randomly selected birds in the front, middle, and back of the barn in an attempt to get a clear overview of the flock. Farmers were asked to consider both older (scabbed) and newer/fresh injuries when scoring their birds. If farmers encountered multiple injuries on the same body area of a bird, they were asked to pick the most severe injury to score. Although clear instructions were given, it should be acknowledged that it was impossible to check and ensure all farmers followed the instructions correctly. However, farmer-reported data is a valid tool in animal welfare research and has been relied on in similar studies (24, 25).

### Survey Distribution

The survey (questionnaire + scoring guide) was distributed *via* the Turkey Farmers of Canada to farmers in all turkey producing provinces in April 2019. Survey components were available in English and French and hard copy or online formats (Qualtrics, Provo, UT), depending on the farmer's preference. Each survey package contained a cover letter explaining the purpose of the study, questionnaire, scoring guide, and pre-paid return envelope with a unique code to ensure the anonymity of responses. Data collection ended in December 2019. This study was approved by the University of Guelph Research Ethics Board (REB 19-02-015) and the University of Guelph Animal Care Committee (AUP 3782).

Out of 500 surveys distributed, 101 responses were received (~20% response rate). Responses were collected from all producing provinces including British Columbia, Alberta, Saskatchewan, Manitoba, Ontario, Quebec, New Brunswick, and Nova Scotia (22). Thirty-eight responses were excluded because they did not complete the entire survey due to no longer producing turkeys, failure to complete the scoring portion, or incorrect interpretation of the instructions. These exclusions left a total of 63 surveys to be included in the analysis (Table 1). All flocks were commercial birds raised for meat production with the exception of four hen flocks (20–39 weeks of age) that were indicated to be breeder flocks.

**Table 1:** Prevalence of pecking injuries from 63 commercial turkey flocks used in this analysis.

	***N***	**Mean (SD)**	**Minimum**	**Maximum**
Head and neck injuries^a^	63			
Toms	24	7.78 (9.10)	0	40.00
Hens	39	5.04 (7.79)	0	36.67
Back and tail injuries^b^	63			
Toms	24	13.61 (19.58)	0	96.67
Hens	39	7.26 (8.98)	0	33.33

a *Any injury (old or new) found on the head, neck, wattle, or snood*.

b *Any injury (old or new) found on the back and tail area*.

### Statistical Analysis

The prevalence of HN and BT injuries was estimated as the number of birds in the scored subset with an injury score greater than 0 (i.e., score 1 or 2) (Table 1). Bird, housing, management, and health characteristics obtained from the questionnaire were used to determine variables associated with the prevalence of HN and BT injuries. Univariable and multivariable models testing the association of the variables with injuries were performed using RStudio (version 3.5.3).

### Data Cleaning

Data collected from the questionnaire (97 variables) were entered into Excel using manual double entry to check and correct potential errors. Variables were excluded from further evaluation if they were missing many responses or did not have enough variation in responses (i.e., a binary variable with >85% of responses in one category). In some cases, variable categories were collapsed retrospectively to remove unused categories. After this step, 53 remained for univariable analyses for HN and BT injuries.

### Univariable Analysis

Due to the low prevalence of HN and BT injuries, flocks were classified as having an issue with the respective pecking injury (1) or not (0), based on a median cut-off value (HN = 3.33%, BT = 6.66%). Univariable logistic regression was then performed to determine which factors were associated with having a prevalence >3.33% of HN injuries or >6.66% of BT injuries.

For the univariable analysis, the variables which were kept for further analysis were required to have a *P* ≤ 0.25. Pearson correlations were determined between the retained variables to test for redundancy. If the correlation between a pair of variables was strong (R > 0.5), only one variable (more relevant or better distribution of responses) was used in the multivariable analysis. After this assessment, 15 variables progressed to the multivariable analysis for HN injuries, and 14 variables progressed for BT injuries.

### Multivariable Analysis

For the multivariable analyses, logistic regression modeling with a backward selection approach based on variable *P*-values was performed for HN and BT injuries. In an iterative process, the variable with the highest *P*-value was removed at each stage until the *P-*values of the model variables were <0.1 (tendencies) and/or contributed to a higher McFadden's pseudo-R^2^ (26). Due to the cross-sectional nature of the study which encompassed tom and hen flocks of varying ages, age and sex were retained in the multivariable model for both traits. Additionally, age and sex positively contributed to the McFadden's pseudo-R^2^ of the models. McFadden's pseudo-R^2^ ranges between 0 and 1 with a value of 1 meaning that the model can predict the outcome with 100% accuracy. A McFadden's pseudo-R^2^ value between 0.20 and 0.40 indicates excellent model fit (27). Results are presented as odds ratio (**OR**) where an OR > 1 indicates that a flock with the response category had higher odds of having a prevalence above the median for the respective injury (i.e., >3.33% HN injuries or >6.66% BT injuries) compared to flocks in the reference category.

## Results

### Flock Information

Of the 63 flocks, 62% were hen flocks (*N* = 39) and 38% were tom flocks (*N* = 24) (Table 1). The hen and tom flocks included in the analysis both had a mean age of 11 weeks (SD_hens_: 7 weeks, SD_toms_= 3 weeks), although toms were heavier on average (9 ± 3.5 kg) than hens (6 ± 3.0 kg). The mean flock size was 8,030 ± 5,833 and 5,885 ± 4,597 birds for hen and tom flocks, respectively.

### Univariable Analysis

Variables associated at the univariable level (*P* ≤ 0.25) with the presence of HN injuries and BT injuries in turkey flocks are presented in Tables 2, 3, respectively.

**Table 2:** Explanatory variables associated (*P* ≤ 0.25) with the presence of head/neck (HN) injuries at the univariable level.

**Variable**	***N* (%)**	**OR^a^**	**95% CI^b^**	***P*-value**
Flock sex				0.10
Hens	39 (61.9)	Referent	Referent	
Toms	24 (38.1)	2.36	0.84–6.86	
Flock breed				0.25
Other	14 (23.3)	Referent	Referent	
Hybrid Converter	46 (76.7)	2.10	0.61–8.56	
Snood removal				0.11
No	44 (72.1)	Referent	Referent	
Yes	17 (27.9)	2.71	0.81–10.82	
Growing system				0.04
Brood to finish	15 (25.0)	Referent	Referent	
Brood and move	45 (75.0)	3.83	1.05–18.48	
Flock age (wks)^c^	63 (100.0)	1.15	1.03–1.32	0.01
Bird weight (kg)^d^	63 (100.0)	1.20	1.03–1.42	0.02
Stocking density (kg/m^2^)	62 (100.0)	1.05	1.01–1.10	0.01
Flooring type				
Dirt	7 (11.7)	Referent	Referent	0.19
Concrete	41 (68.3)	1.04	0.21–5.86	
Combination	12 (20.0)	0.27	0.03–2.19	
Light intensity				0.18
≤ 20 lux	33 (66.0)	Referent	Referent	
> 20 lux	17 (34.0)	2.25	0.68–7.65	
Intermittent lighting				0.14
Yes	12 (19.7)	Referent	Referent	
No	49 (80.3)	0.38	0.10–1.36	
Ventilation type				0.05
Power	39 (61.9)	Referent	Referent	
Natural	8 (12.7)	6.75	1.34–50.89	
Mixed	16 (25.4)	2.25	0.68–7.58	
Feed/water additives				0.15
Yes	10 (16.4)	Referent	Referent	
No	51 (83.6)	0.36	0.08–1.44	
Number of daily inspections				0.06
2 times or less	35 (55.6)	Referent	Referent	
More than 2 times	28 (44.4)	0.38	0.13–1.06	
Number of people inspecting				0.06
1 person	23 (36.5)	Referent	Referent	
More than 1 person	40 (63.5)	2.83	0.96–9.23	
Duration of inspections				0.19
≤ 30 min	51 (81.0)	Referent	Referent	
> 30 min	12 (19.0)	2.36	0.66–8.98	
Picking up birds during inspections				0.05
Never/sometimes	28 (45.2)	Referent	Referent	
Half of the time or more	34 (54.8)	0.361	0.12–1.01	

a *Odds ratio (OR)*.

b *95% confidence interval (CI)*.

c *Continuous variable. OR represents increase in odds with every unit (wk) increase in bird age*.

d *Continuous variable. OR represents increase in odds with every unit (kg) increase in bird weight*.

**Table 3:** Explanatory variables associated (*P* ≤ 0.25) with the presence of back/tail (BT) injuries at the univariable level.

**Variable**	***N* (%)**	**OR^a^**	**95% CI^b^**	***P*-value**
Flock sex				0.05
Hens	39 (61.9)	Referent	Referent	
Toms	24 (38.1)	2.80	0.99–8.23	
Toe trimming				0.02
No	29 (47.5)	Referent	Referent	
Yes	32 (52.5)	0.276	0.09–0.079	
Claw trimming				0.22
No	47 (77.0)	Referent	Referent	
Yes	14 (23.0)	0.45	0.11–1.57	
Snood removal				0.03
No	44 (72.1)	Referent	Referent	
Yes	17 (27.9)	3.54	1.13–12.12	
Growing system				0.04
Brood to finish	15 (25.0)	Referent	Referent	
Brood and move	45 (75.0)	3.83	1.05–18.48	
Flock age (wks)^c^	63 (100.0)	1.12	1.01–1.30	0.05
Bird weight (kg)^d^	63 (100.0)	1.14	0.98–1.33	0.09
Stocking density (kg/m^2^)	62 (100.0)	1.03	0.99–1.07	0.17
Litter depth (cm)^e^	61 (100.0)	1.33	0.89–2.05	0.18
Litter tilling				
No	52 (85.2)	Referent	Referent	0.12
Yes	9 (14.8)	3.20	0.76–16.54	
Feed Structure				0.16
Mash or crumbs	12 (19.7)	Referent	Referent	
Pellets	49 (80.3)	2.65	0.70–13.05	
Drinker type				0.17
Closed	44 (71.0)	Referent	Referent	
Open	18 (29.0)	2.19	0.72–6.85	
Inspection duration				0.06
≤ 30 min	51 (81.0)	Referent	Referent	
> 30 min	12 (19.0)	3.37	0.93–14.06	
Picking up birds				0.24
Never or sometimes	28 (45.2)	Referent	Referent	
Half of the time or more	34 (54.8)	0.55	0.19–1.51	
Use hospital pens				0.21
Yes	23 (37.1)	Referent	Referent	
No	39 (62.9)	0.51	0.18–1.46	

a *Odds ratio (OR)*.

b *95% confidence interval (CI)*.

c *Continuous variable. OR represents increase in odds with every unit (wk) increase in bird age*.

d *Continuous variable. OR represents increase in odds with every unit (kg) increase in bird weight*.

e *Continuous variable. OR represents increase in odds with every unit (cm) increase in litter depth*.

### Multivariable Analysis

The final logistic regression model for the presence of HN injuries included five variables and accounted for ~23% of the variation in the presence of HN injuries (*N* = 62, pR^2^ = 0.23, α = 0.05, Table 4). The variables included in the final model were flock sex, flock age, number of daily inspections, number of people inspecting the flock, and picking up birds during inspections. Flock sex was retained in the final model because of its biological relevance and contribution to the McFadden's pseudo-R^2^, although there was no difference in the presence of HN injuries between tom and hen flocks (OR = 2.02, 95%CI: 0.59–7.08, *P* = 0.15). The odds of having a prevalence of HN injuries above the median (>3.33%) within a flock increased as flocks got older (OR = 1.11, 95%CI: 0.99–1.29, *P* = 0.02). Interestingly, the remaining variables retained in the final model relate to different aspects of the daily inspections of the flock. Flocks that were inspected more frequently (more than twice daily) tended to decrease the likelihood of having HN injuries compared to flocks inspected two times or less per day (OR = 0.22, 95%CI: 0.05–0.79, *P* = 0.08). However, flocks that were inspected daily by more than one person were 4x more likely to have HN injuries compared to flocks which were only inspected by one person (OR = 4.05, 95%CI: 1.07–18.52, *P* = 0.05). Lastly, picking up birds during the inspections tended to increase the odds of having HN injuries (OR = 3.30, 95%CI: 0.984–12.400, *P* = 0.05).

**Table 4:** Final logistic regression model for the presence of HN injuries (*N* = 62, pR^2^ = 0.23, alpha = 0.05).

**Variable**	**OR^a^**	**95% CI^b^**	***P*-value**
Flock sex			0.15
Hens	Referent	Referent	
Toms	2.02	0.59–7.08	
Flock age^c^	1.11	0.99–1.29	0.02
Number of daily inspections			0.08
Two times or less	Referent	Referent	
More than two times	0.22	0.05–0.79	
Number of people inspecting			0.05
One person	Referent	Referent	
More than one person	4.05	1.07–18.52	
Picking up birds			0.05
Never or sometimes	Referent	Referent	
Half the time or more	3.30	0.98–12.40	

a *Odds ratio (OR)*.

b *95% confidence interval (CI)*.

c *Continuous variable. OR represents increase in odds with every unit (wk) increase in bird age*.

The final logistic regression model for the presence of BT injuries explained approximately 29% of the variation in the presence of BT injuries (N = 59, pR^2^ = 0.29, α = 0.05, Table 5) and included flock sex, flock age, litter depth, litter condition, duration of inspections, and the use of hospital pens. In this case, the odds of having BT injuries were different between the sexes. Tom flocks were 4x more likely to have BT injuries than hen flocks (OR = 4.03, 95%CI: 1.14–15.96, *P* = 0.02). As flocks got older, the odds of having BT injuries tended to increase (OR = 1.06, 95%CI = 0.93–1.23, *P* = 0.05) which is similar to HN injuries. Flocks that had poor litter condition (dusty or damp) tended to have more BT injuries compared to flocks that had good litter condition (OR = 3.49, 95%CI = 0.94–14.60, *P* = 0.09). Finally, using hospital pens tended to increase the odds of having BT injuries (OR = 3.86, 95%CI = 0.97–17.72, *P* = 0.05).

**Table 5:** Final logistic regression model for the presence of BT injuries (*N* = 59, pR^2^ = 0.29, alpha = 0.05).

**Variable**	**OR^a^**	**95% CI^b^**	***P*-value**
Flock sex			0.01
Hens	Referent	Referent	
Toms	4.03	1.14–15.96	
Flock age^c^	1.06	0.93–1.23	0.05
Litter depth (cm)^d^	1.71	0.97–3.18	0.19
Litter condition			0.09
Good condition	Referent	Referent	
Dusty/damp litter	3.49	0.94–14.60	
Duration of inspections			0.12
≤ 30 min	Referent	Referent	
> 30 min	3.70	0.75–23.27	
Use hospital pens			0.05
No	Referent	Referent	
Yes	3.86	0.97–17.72	

a *Odds ratio (OR)*.

b *95% confidence interval (CI)*.

c *Continuous variable. OR represents increase in odds with every unit (wk) increase in bird age*.

d *Continuous variable. OR represents increase in odds with every unit (cm) increase in litter depth*.

## Discussion

This study aimed to identify factors associated with the prevalence of injuries in the HN and BT area in turkey flocks as they may reflect different behavioral motivations. A questionnaire and flock injury scoring were completed by turkey farmers across Canada to gather information about housing and management characteristics and the presence of injuries in commercial flocks. For the logistic regression analysis, the prevalence of HN and BT injuries in each flock was classified into a binary variable based on the median prevalence of the injuries in the different areas (HN = 3.3%, BT = 6.7%). Variables related to flock characteristics, housing, and management were obtained from the questionnaire and tested for their association with pecking injuries to both body areas.

The median prevalence of HN injuries was 3.33% (mean: 6.1%, range: 0.0–40.0%), and the median prevalence of BT injuries was 6.7% (mean: 9.7%, range: 0.0–96.7%) which indicates that on average one or two birds were affected out of the 30 sampled. While the median prevalence for these conditions was relatively low, there was a large range between flocks. Typically, HN injuries are recorded less frequently than BT injuries (7, 21, 28). In a study of 60 turkey flocks in France (100–300 samples per flock), Allain et al. (28) reported an average of 6.6% of birds within a flock with feather pecking injuries at the processing plant, and the average prevalence of head injuries was much lower (0.1%). The present study reports a higher average prevalence of HN injuries (6.1%), but this could be attributed to the varying flock ages and cross-sectional study design or differences in scoring scales. This finding could also be the result of the small subset of birds scored in this study since the minimum prevalence that could be reported by a farmer was 3.33% (one bird affected). However, other studies have reported the mean prevalence of general skin injuries in turkeys as high as 31.0%, although there was no differentiation between body areas (29). The large range in prevalence found in this study also points to environmental conditions and/or management having a large influence on prevalence.

The variables identified from this study require further longitudinal research to truly determine their influence on pecking injuries. However, this is an important starting point for exploring contributing factors to injurious pecking in North American turkey production systems.

### Risk Factors Associated With Head/Neck Injuries

The variables in the final model for HN injuries relate to how the flock was inspected, including the number of daily inspections, number of people inspecting, and picking up birds during inspections. Good stockmanship and flock inspections are critical components of animal welfare and productivity (30–32). The Canadian Code of Practice describes inspections as the process of routinely checking flocks and/or barns for parameters surrounding bird health, wellbeing and access to feed and water (33).

Interestingly, flocks which were inspected by multiple people within the same day were 4x more likely to have HN injuries compared to flocks that were only inspected by one person. Assigning one person to a flock may be beneficial by creating a sense of ownership in that flock's performance and this person may feel more responsible for ensuring bird welfare. This could result in that one person being very diligent in the barn biosecurity, maintenance, and flock inspection as they are solely responsible whereas with multiple people there is less accountability. Moreover, the presence of familiar humans has been linked to lower levels of distress and risk of injury to both animals and humans (31, 32). However, studies of human-avian interactions in poultry, especially turkeys, are lacking. Chicks are well known for imprinting and can recognize familiar objects and have a demonstrated preference for face-like stimuli over featureless stimuli (34, 35). Moreover, wild bird species are capable of recognizing individual humans (36–39). It is possible that having the same person inspect the turkey flock allows birds to become familiar with that stockperson, and thus be less likely to be distressed during inspections. Injurious pecking has been observed to be initiated after a “general arousal” in a turkey flock without any other noticeable cause (5).

Additionally, in flocks where birds were picked up more frequently (half the time or more), the odds of having HN injuries tended to be higher. While regularly handling animals during inspections has been demonstrated to have beneficial effects in turkeys [e.g., reduction in prevalence of footpad dermatitis, (40)], there may be negative consequences for aggressive pecking. It has been demonstrated that reactivity to manual restraint has a relationship with severe feather pecking behaviors in laying hens (41, 42). While this variable was not included in the final model for BT injuries, at the univariable level a similar relationship was found where handling the birds less decreased the odds of BT injuries. Therefore, it is possible that frequent handling can also have negative consequences for injurious pecking if the birds perceive it as a source of stress. We are also assuming in this analysis that proper turkey handling technique is being used on every farm. Most farmers who responded to the survey had more than 10 years of experience in turkey production (22), however, this does not guarantee that the staff on each farm had the same training and experience. Improper handling technique, especially if being handled many times, may be more stressful for the birds and may increase pecking or injuries. Based on the inclusion of several human-related traits in the final model for HN injuries, clarifying how human presence influences aggression and injuries may assist in reducing these problems.

Finally, flocks must be inspected at least twice daily, but it is recommended to increase the frequency for better health monitoring (33). Flocks that were inspected more frequently tended to have lower odds of having HN injuries. This potentially indicates the importance of the stockperson in reducing aggressive pecking and injuries by acclimating animals to human exposure, which reduces stress and aggressive behaviors (43). Increased inspection frequency may also be a sign that the farmer is more concerned with the flock's wellbeing and this is reflected in better management practices for other husbandry aspects (e.g., veterinary care, litter management, air quality). On the other hand, it is possible that with more frequent inspections, birds with injuries may be culled more often, reducing the number of birds with injuries in the flock. The presence of injuries or bleeding can escalate pecking into cannibalism (2), and potentially propagate the problem through the flock (44). Pecking injuries and cannibalism were reported as a reason for culling and mortality by nearly 40% of the farmers in this study (13). Less frequent inspections may leave injured birds in the flock longer, potentially being the reason for the higher prevalence of HN injuries observed. However, the decision to cull birds in a timely manner is dependent on cull plans, clearly defined end-points, training and farmer perception (45).

### Risk Factors Associated With Back/Tail Injuries

Factors related to litter management and general flock care were included in the final model for BT injuries, including litter condition, litter depth, duration of inspections, and use of hospital pens. Some poultry farmers use hospital pens to segregate injured or ill birds for easier inspection and monitoring (23). These pens provide opportunities for birds to recuperate in semi-isolation before being returned to the flock or euthanized if recuperation is not possible (23). The opportunity for recovery in the absence of flock mates may be especially beneficial in the case of injurious pecking. This allows the victim a chance to escape the aggressor(s) and recover from injuries before it escalates to cannibalism. We expected farmers who used these pens to have lower odds of having BT injuries; however, we found when these pens were used birds tended to be 3.9x more likely to have BT injuries. It could be that the removal, isolation, and then reintroduction of birds from the flock actually results in more frequent disruptions of the social hierarchy. Social mixing is considered stressful for poultry which could increase the frequency of pecking behaviors which may explain why we saw greater injuries in the group using hospital pens (46). It is important to note that this association was only a tendency and may be a response to a pecking outbreak or farmers who habitually have problems with feather pecking in their flocks. Similarly, it could be that farmers spent more time during inspections when problems in flocks are already present, hence explaining the equal trend for more BT injuries in flocks where inspections lasted longer. Due to the nature of the survey, we cannot know if the farmer always used hospital pens/took more time for inspections or if they implemented these practices in response to a problem. If it is in response to a problem, it is a positive sign that farmers recognize the potential negative effects of leaving injured birds in the flock and segregating birds accordingly.

Proper litter management can positively influence bird health and behavior by contributing to excreta breakdown, aiding in moisture evaporation, and maintaining friable litter that birds can “work” (47). Subjective assessment of the litter condition indicated that litter that was too dusty or conversely too damp pointed toward issues with BT injuries, though this relationship was not significant in this study. Similarly, litter depth was included in the final model but did not significantly affect the odds of having BT injuries similar to previous work in chickens (48, 49). Loose friable litter of adequate depth is recommended (33), increases opportunities for foraging and exploratory behaviors (48), and good litter management is thought to be one of the main factors in preventing or reducing feather pecking (9, 50). Due to conflicting results in the literature, further research is needed in this area to properly define the characteristics of good litter quality and how to maintain it to assess its impact on injurious pecking in turkeys.

### Effect of Age on HN and BT Injuries

Aggressive behavior and injuries from aggression are commonly reported to increase with age (7, 21). As shown in Table 4, with every 1-week increase in age, flocks were 1.11x more likely to have HN injuries. While this is a small increase in likelihood, it is important to note that aggressive pecking can appear quite early in life for toms (20–30 days), so the cumulative effect over the production period of the bird may be substantial (6, 44). In wild turkey populations, aggressive pecking to form stable hierarchies does not result in serious injuries because there is sufficient space for birds to avoid each other (5). In the case of domestic turkey flocks, there is less opportunity to effectively escape aggressors, and individual identification is more difficult, which can prevent the formation of a stable hierarchy, leading to continued aggressive interactions (5). Martrenchar et al. (51) found that aggressive pecking increased between 5 and 10 weeks of age for both toms and hens, although the increase was more substantial in toms. In line with this increase in aggressive behavior, there was also a corresponding increase in the incidence of head injuries (51).

Furthermore, pecking behavior (both aggressive and feather pecking) is socially transmissible, meaning that birds can learn the behavior from their flock mates (44). This implies a potential exponential increase in pecking behavior as more and more birds in the flock are exposed to, and learn, this behavior over time. Bartels et al. (5) noted anecdotally that fighting between birds attracted the attention of the other birds in the flock such that defeated turkeys would be pecked by the original aggressor and by bystanders. Although the frequency of injurious pecking behavior increases as birds age, the duration of the actual pecking bouts, that result in serious injury, decreases (5). This may mean that pecks are more forceful when birds are older, leading to a higher likelihood of serious injury resulting in mortality or culling (5).

Like aggressive pecking, severe feather pecking behavior (which typically targets the BT area) increases with age (52). Accompanying the behavior, feather damage and injuries from feather pecking also increase as birds get older (21). However, due to the study's cross-sectional nature, we cannot determine how injuries develop in a turkey flock over time. Conversely, Busayi et al. (52) found that general pecking behavior in turkeys decreases from 3 to 9 weeks of age. Unfortunately, the study of Busayi et al. (52) ended at 9 weeks of age and commercial turkeys are typically slaughtered between 9 and 20 weeks depending on the sex and desired market weight. Duggan et al. (12) assessed feather and skin damage in turkeys from 6 to 15 weeks of age in different housing situations. They found that the damage score worsened over time in curtain-sided barns, but the damage score actually improved in mechanically-controlled housing (12). These findings emphasize the importance of the environment in controlling injurious pecking.

Aside from environmental and social factors, it is also possible that the probability of getting pecked simply increases over time. Further longitudinal studies in this area are needed for Canadian turkey flocks to properly determine how feather pecking develops and spreads over time, especially considering different types of housing and management conditions.

### Effect of Sex on HN and BT Injuries

Aggressive pecking with resulting head injuries is typically more common in toms as this behavior relates to dominance and establishing social hierarchy (51–55). However, we found no difference in the odds of having HN injuries between tom and hen flocks. It is possible that we did not find a difference between the sexes due to the cross-sectional nature of our study and variety of ages, flock sizes etc. between the two sexes. Furthermore, 30 birds were chosen as a scoring sample because it was determined to be feasible for the farmer to complete in a reasonable time frame. We cannot exclude the possibility that tom flocks may have been more aggressive than hen flocks, but there might not have been an observable difference in the prevalence of the injury within the sample of birds scored. Birds with clearly observable head wounds (expected more in tom flocks) may be culled more frequently or sequestered away from the rest of the flock and may not have been chosen for scoring despite instructions to select birds randomly.

We found that toms flocks were more likely to have BT injuries compared to hen flocks. Martrenchar et al. (51) found that toms removed 2-3x more feathers than hens at 10 weeks of age, despite hens performing 2x more pecking behavior than toms. Busayi et al. (52) also reported that toms exhibit stronger feather pecks and pulls at 3 weeks of age compared to hens. These results indicate that tom pecks may be more forceful and more likely to cause damage, which might be why there tends to be greater pecking injuries in tom flocks, even if hens express the behavior more. However, there is no clear consensus in the literature whether feather pecking is more common in toms or hens, and this should be explored further, especially under commercial conditions.

This study was an initial, exploratory assessment of factors associated with integument injuries in Canadian turkey flocks, as such the goal was to include as many farms as possible across the entire country. Due to the large distance between farms, it was not feasible for the research team to perform the flock scoring and so this analysis relied on self-reported injury scores from individual farmers. We can, therefore, not discount the possibility that the interpretation of the scoring system was different between farmers. To minimize this possibility, the scoring system was pilot tested by industry stakeholders and based on previous assessment protocols for turkeys (56). As this is a cross-sectional study, the associations and *P*-values presented here are exploratory. More work is needed to design longitudinal studies to better understand and validate the associations identified here.

## Conclusion

We observed HN and BT injuries, respectively, in ~41 and 43% of surveyed Canadian turkey flocks. Injury presence was defined as a flock level prevalence greater than the median prevalence of HN (3.3%) and BT (6.7%) injuries. This indicates that injurious pecking is still a persistent problem on turkey farms, especially considering the relatively young flock ages. The variation in the presence of HN and BT injuries, ~23 and 29%, respectively, were explained by models including different farm management factors and flock sex and age. The odds of BT injuries were greater in tom flocks and the odds of both HN and BT injuries increased with flock age. The final model for HN injuries included the explanatory management variables of number of daily inspections, number of people inspecting, and picking up birds during inspections. The final model for BT injuries included management variables related to litter condition and use of hospital pens. The results from this study indicate that human-animal interaction (e.g., flock inspections and handling) may play a role in the development of HN injuries. In contrast, management factors and foraging opportunities (e.g., hospital pens and litter condition) may influence BT injuries. The associations identified in this study lay the foundation for further research to elucidate causative factors which can help inform housing and management to reduce pecking-related injuries in turkeys.

## Data Availability Statement

The raw data supporting the conclusions of this article will be made available by the authors, without undue reservation.

## Ethics Statement

This study was approved by the University of Guelph Research Ethics Board (REB 19-02-015). The patients/participants provided their written informed consent to participate in this study. The animal study was reviewed and approved by University of Guelph Animal Care Committee (AUP 3782). Written informed consent was obtained from the owners for the participation of their animals in this study.

## Author Contributions

EL, NS, VO, BW, AH-M, and CB conceived and designed the study. EL and NS conducted the study. EL analyzed the data and wrote the main manuscript. All authors reviewed and approved the final manuscript.

## Funding

This project was funded by the Government of Canada through Genome Canada and the Ontario Genomics Institute (OGI-133). This study was part of the project entitled “Application of genomic selection in turkeys for health, welfare, efficiency and production traits” funded by the government of Canada through the Genome Canada Genomic Application Partnership Program and administered by Ontario Genomics [recipients: BW (Industry) and CB (Academic)]. The authors would also like to acknowledge NSERC and Hybrid Turkeys for financial support.

## Conflict of Interest

BW was an employee of Hybrid Turkeys at the time of the study. Hybrid turkeys disseminated an invitation to voluntarily participate in the study amongst its farm managers. The remaining authors declare that the research was conducted in the absence of any commercial or financial relationships that could be construed as a potential conflict of interest.

## Publisher's Note

All claims expressed in this article are solely those of the authors and do not necessarily represent those of their affiliated organizations, or those of the publisher, the editors and the reviewers. Any product that may be evaluated in this article, or claim that may be made by its manufacturer, is not guaranteed or endorsed by the publisher.
