# Supplementary material for: The Prevalence of Integument Injuries and Associated Risk Factors Among Canadian Turkeys
Source: Front Vet Sci. 2022 Jan 7;8:757776. doi: 10.3389/fvets.2021.757776 (PMC8777054; doi:10.3389/fvets.2021.757776)
Supplement: Supplementary file 1 [file Data_Sheet_1.PDF]

A risk factor analysis of health traits in turkeys on Canadian farms

Turkey farms
Risk factor questionnaire

Please fill in the following questions to the best of your ability. Please print clearly when writing down your answers or tick the appropriate box as ☒. Only provide one answer (i.e. tick one box) for each question, unless otherwise indicated. There are no right or wrong answers. If you would like to revise your answer, please draw a single horizontal line through the first answer and give your new answer as instructed. If at any point you need more space, you can use a blank sheet and clearly indicate to which question it relates. Thank you for participating!

Page Symbols:

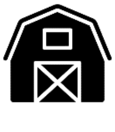

If you see this symbol on the page, answer the questions about your <u>entire farm</u>.

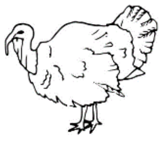

If you see this symbol on the page, answer the questions about a <u>single flock</u> of birds that you choose. Once you have chosen a flock, all pages with this symbol will be answered for that specific flock. You will assess the health scores of these birds.

1. Background information

Please answer questions 1 – 14 about you or your entire farm. Questions 15 – 93 refer to one chosen flock on your farm.

Please fill in the current date (dd/mm/yy):____

1. Please indicate your sex:

   ☐ Female ☐ Male ☐ My sex is not listed ☐ Prefer not to answer

2. Please indicate your gender:

   ☐ Woman ☐ Man ☐ My gender is not listed ☐ Prefer not to answer

3. Please indicate your age:

   ☐ 18-24 ☐ 25-34 ☐ 35-44
   ☐ 45-54 ☐ 55-64 ☐ 65 and above
   ☐ Prefer not to answer

4. Please indicate in which province/territory your farm is located:

   ☐ British Columbia ☐ Quebec ☐ Newfoundland and Labrador
   ☐ Alberta ☐ Yukon ☐ Prince Edward Island
   ☐ Saskatchewan ☐ Northwest Territories ☐ New Brunswick
   ☐ Manitoba ☐ Nunavut ☐ Nova Scotia
   ☐ Ontario ☐ I don't know ☐ Prefer not to answer

5. Please indicate how many year(s) of experience you have working with turkeys:

   ☐ Less than 1 year ☐ 1-4 years ☐ 5-10 years
   ☐ More than 10 years ☐ I don't know ☐ Prefer not to answer

6. Have you completed specific turkey related training course(s) or seminar(s)?

   ☐ Yes – 1 course/seminar ☐ Yes – 2 to 3 courses/seminars
   ☐ Yes – more than 3 courses/seminars ☐ No
   ☐ I don't know ☐ Prefer not to answer

7. Have you or someone on your farm been certified to perform euthanasia by a veterinarian or other professional?

☐ Yes ☐ No
☐ I don’t know ☐ Prefer not to answer

8. Please indicate how often a veterinarian visits your farm:

☐ Once per year
☐ More than once per year
☐ Only as needed
☐ I don’t know
☐ Prefer not to answer

9. Do you have an existing relationship with your veterinarian (VCPR)?

☐ Yes ☐ No ☐ I don’t know ☐ Prefer not to answer

10. How many barns (a barn is defined as a building that houses one flock) are on your farm?

☐ 1 ☐ 2 ☐ 3 ☐ 4 ☐ 5 ☐ More than 5
☐ I don’t know ☐ Prefer not to answer

11. Do you have a single age class or multiple age classes on your farm?

☐ Single age farm ☐ Multi-age farm ☐ I don’t know ☐ Prefer not to answer

12. Please give the total number of turkeys currently on your farm: ___________________

13. How many turkeys do you typically raise per year on your farm? __________________

14. Please indicate the typical type of turkey production on your farm: *Select all that apply*

☐ Broilers (6.2 kg and under)
☐ Light hens (up to 7.5 kg)
☐ Heavy hens (7.5 to 10.0 kg)
☐ I don’t know
☐ Prefer not to answer
☐ Light toms (10.0 kg and under)
☐ Heavy toms (more than 10.0 kg)
☐ Mature birds

2. Bird Characteristics – Chosen Flock

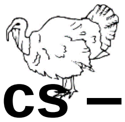
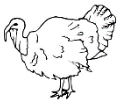

Please answer the following questions for <u>one barn</u>, which houses one flock of birds. If you have multiple barns on your farm, please choose one barn and answer the following questions about the same flock. This is also the barn where you will <u>assess the birds' health scores</u>.

15. Please indicate the sex of the birds in the chosen flock.

☐ Hens ☐ Toms ☐ Mixed
☐ I don't know ☐ Prefer not to answer

16. What company/hatchery are the birds sourced from?

☐ Cuddy ☐ Other. Please specify:___________________________
☐ Cooper Farms ☐ I don't know
☐ Maple Leaf ☐ Prefer not to answer
☐ La Coop Fédérée
☐ Lilydale

17. Please indicate the specific strain(s) of birds in the chosen flock. *Select all that apply*

☐ Hybrid Converter ☐ Other. Please specify:_____________________
☐ Aviagen B.U.T. 6 ☐ I don't know
☐ Aviagen Nicholas Select ☐ Prefer not to answer
☐ Orlopp Bronze
☐ MiniClassic
☐ Artisan Gold

18. Have these birds undergone any physical alterations in the hatchery? *Select all that apply.*

☐ Beak treatment ☐ Toenail treatment ☐ Dew claw removal
☐ Snood removal ☐ I don't know ☐ Prefer not to answer

19. If the chosen flock is beak trimmed, do you have any problems with the quality of the trim? (i.e., trim too short, trim too long, trim has rough/jagged edges, deformities, etc.)

☐ Yes. Please specify the problem:____
☐ No ☐ I don't know ☐ Prefer not to answer ☐ Does not apply

20. At what date were the birds placed in their current barn (dd/mm/yy)?_________________

21. Where was the chosen flock brooded?

☐ In the current barn (brood to finish growing system).
☐ In a brooding barn (brood and move growing system).
☐ I don't know
☐ Prefer not to answer

22. When the birds arrived from the hatchery, did you notice a large variation in body size at placement?

☐ Yes ☐ No ☐ I don't know ☐ Prefer not to answer

23. When the birds were moved from the brooding to the grow-out barn, did you notice any of the following? *Select all that apply.*

☐ Does not apply
☐ Birds with varying body size
☐ Birds with leg injuries (i.e. broken legs, footpad dermatitis)
☐ Birds with leg deformities (i.e. cowboy legs, hockey stick leg, splay leg)
☐ Birds with blood visible on their body
☐ Birds with feather damage/naked spots
☐ Birds with wing injuries (i.e. broken wings)
☐ Other. Please specify:___________________________________
☐ None of the above ☐ I don't know ☐ Prefer not to answer

24. What is the current age of the birds in the barn (weeks)?__________________________

25. How many birds were placed in the barn in total?________________________________

26. How many birds are currently in the barn?_____________________________________

27. Please indicate the current stocking density at which the birds are housed*:

______________birds/m^2 ______________kg/m^2

______________lbs/sqft ______________birds/sqft

**Please answer with your preferred unit of measurement

28. Have you needed to perform any physical alterations (i.e. beak trimming) after the placement of the chosen flock on your farm?

☐ Yes. Please specify which alteration(s) was performed:________________________
Please specify why the alteration(s) was performed:_______________________
☐ No ☐ I don't know ☐ Prefer not to answer

29. What is the current approximate weight of the birds in the chosen barn?
____________________lbs or kg (*please circle unit of measurement)*

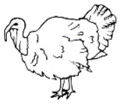

30. What is the approximate finishing weight of the birds in the chosen barn?
____________________lbs or kg (*please circle unit of measurement*)

31. Is this flock certified in any of the following? *Select all that apply*

☐ Organic
☐ Raised Without Antibiotics (RWA) / Antibiotic Free (ABF)
☐ Responsible Use of Antibiotics (RUA)
☐ Other. Please specify:____
☐ I don't know
☐ Prefer not to answer

3. Housing – Chosen Flock 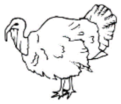

32. What is the square footage of the space that houses the chosen flock?____________sqft

33. Please indicate the housing system of the barn:

☐ Natural (open-sided housing with curtains) Please specify curtain colour:__________
☐ Power (controlled environment housing)
☐ Other. Please specify:____
☐ I don’t know ☐ Prefer not to answer

34. Does the barn which houses the chosen flock have more than 1 storey?

☐ Yes ☐ No ☐ I don’t know ☐ Prefer not to answer

35. Please indicate the flooring material of the barn which houses the chosen flock:

☐ Dirt
☐ Wood
☐ Concrete
☐ Other. Please specify:____
☐ I don’t know ☐ Prefer not to answer

36. Please indicate the number of years since construction or last major renovation of the barn:

☐ Less than 1 year ☐ 1-4 years ☐ 5-10 years
☐ More than 10 years ☐ I don’t know ☐ Prefer not to answer

37. Do you provide enrichment as a routine practice?

☐ Yes
☐ No. Only in response to aggressive or abnormal behaviour.
☐ No. I never provide enrichment.
☐ I don’t know
☐ Prefer not to answer

38. Has enrichment been provided to the chosen flock?

☐ Yes ☐ No ☐ I don’t know ☐ Prefer not to answer

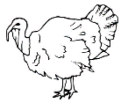

39. If you provided enrichment to this flock, what kind of enrichment did you provide? *Select all that apply*

☐ Does not apply
☐ Bales of hay/straw on ground
☐ Hay/straw hanging in nets
☐ Hanging objects (e.g. aluminum pie plate, CDs)
☐ Objects on the ground (e.g. balls)
☐ Perches
☐ Other. Please specify:____
☐ I don't know
☐ Prefer not to answer

Lighting 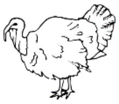

40. Please indicate which type of lighting is provided in the barn: *Select all that apply*

☐ Incandescent ☐ Fluorescent
☐ Natural light (i.e. windows) ☐ LED
☐ Other. Please specify:____
☐ I don't know ☐ Prefer not to answer

41. Please indicate what the average light intensity is (lux) at bird height:

☐ Less than 10 lux ☐ 10-20 lux ☐ 21-30 lux
☐ More than 30 lux ☐ I don't know ☐ Prefer not to answer

42. Is light intensity even throughout the barn (i.e. no darker areas or bright spots)?

☐ Even ☐ Somewhat even ☐ Somewhat uneven
☐ Uneven ☐ I don't know ☐ Prefer not to answer

43. Do you provide a dark period for the chosen flock at their current age?

☐ Yes. Please specify the length of the dark period in hours:_____________________
☐ No ☐ I don't know ☐ Prefer not to answer

44. Do you provide an intermittent light program for the chosen flock at their current age?

☐ Yes. Please specify the number of dark periods in 24 hours:________________
☐ No ☐ I don't know ☐ Prefer not to answer

45. Have you needed to reduce light intensity to correct abnormal or aggressive behaviours in the chosen flock?

☐ Yes ☐ No ☐ I don't know ☐ Prefer not to answer

Air quality 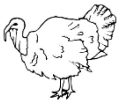

46. Please indicate what type of ventilation system is used:

☐ Mechanical (fans)
☐ Mixed (fans and natural)
☐ I don't know
☐ All natural
☐ Other. Please specify:______________
☐ Prefer not to answer

47. If mechanical ventilation is used, please specify the method of mechanical ventilation:

☐ Cross flow ventilation
☐ Tunnel flow ventilation
☐ Cross and tunnel flow hybrid
☐ Other. Please specify:____
☐ I don't know
☐ Prefer not to answer

48. Which phrase best describes your ventilation system within the barn?

Fresh air inlets:

☐ Side wall(s)
☐ End wall(s)
☐ Attic
☐ Other. Please specify:____
☐ I don't know
☐ Prefer not to answer

Exhaust:

☐ Side wall(s)
☐ End wall(s)
☐ Chimney stacks
☐ Other. Please specify:____
☐ I don't know
☐ Prefer not to answer

49. What is the minimum ventilation rate (in the case of mechanical ventilation) in the chosen barn?_________________________CFM (cubic feet per minute)

50. Please indicate the target temperature in the barn for the birds at their current age*:
___________°C
___________°F
*Please answer with your preferred unit of measurement

51. Please indicate the target relative humidity (%RH) in the barn:_______________

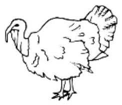

52. Has the concentration of ammonia or dust ever been above 20-25 ppm, i.e. irritable to workers’ eyes or airways, since placement of birds in the barn?

☐ Yes ☐ No ☐ I don’t know ☐ Prefer not to answer

Litter management 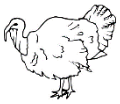

53. What type of litter is provided to the chosen flock? *Select all that apply*

☐ Sand ☐ Straw
☐ Wood shavings. Please specify what kind:___________________________________
☐ No litter provided ☐ Other. Please specify:___________________________
☐ I don't know ☐ Prefer not to answer

54. What approximate depth is the litter currently*?_________inch _____________cm
*Please answer with your preferred unit of measurement

55. Please indicate the current condition of the litter:

☐ Litter very dry and dusty
☐ Low moisture content, litter is loose in all the house
☐ Litter damp or tacky (litter and faecal material sticks to feet, but some dry areas)
☐ Majority of litter is wet, sticky, or greasy (bird's feathers likely to be soiled)
☐ Soggy, squelchy, or very wet (leaves a durable imprint when compressed)
☐ Other. Please specify:____
☐ I don't know
☐ Prefer not to answer

56. How do you check litter condition of the chosen flock? *Select all that apply*

☐ Test with heel ☐ Do not check litter condition
☐ Feel litter with hand ☐ I don't know
☐ Smell the litter ☐ Prefer not to answer
☐ Visual assessment ☐ Other. Please specify:_________________

57. How do you maintain litter quality in the chosen flock? *Select all that apply*

☐ Wet and/or caked litter is removed regularly
☐ Dry litter is added over wet areas as needed.
Please specify bedding type added (i.e., wood shavings, straw, etc.):_______________
☐ Sprinklers used to avoid dusting
☐ Heat is added in the case of wet litter to avoid caking
☐ Tilling the litter
☐ Move the waterers often to prevent wet areas (donuts) from forming
☐ Litter treatment products (e.g., PLT, algae, biomaterials). Please specify:__________
☐ Other. Please specify:____
☐ I don't know
☐ Prefer not to answer

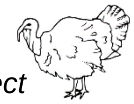

58. If you add more bedding, what area of the barn do you typically add more litter to? *Select all that apply*

☐ Not applicable ☐ Around the water lines ☐ Between water and feed lines
☐ Around perimeter ☐ Entire barn ☐ I don't know
☐ Prefer not to answer
☐ Other. Please specify:____

59. Have previous flocks been raised on the litter currently in the barn?

☐ Yes. Please specify how many flocks: ____
☐ No ☐ I don't know ☐ Prefer not to answer

60. Do you add any products to the feed or water to reduce wetness of the litter?

☐ Yes. Please specify:____
☐ No ☐ I don't know ☐ Prefer not to answer

Feeding 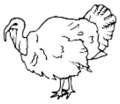

61. Please indicate the number of birds per feed pan in the barn:______________________

62. Please indicate the structure of the feed you currently give the birds:

☐ Mash
☐ Crumbs
☐ Pellets
☐ Other. Please specify:____
☐ I don't know
☐ Prefer not to answer

63. How many times have you changed the diet of the chosen flock since placement on your farm?

☐ No change ☐ 1 – 2 times ☐ 3 – 4 times
☐ 5 – 6 times ☐ 7 – 8 times ☐ More than 8 times
☐ I don't know ☐ Prefer not to answer

64. What is the source of the feed?

☐ Home-milled
☐ Purchased from supplier:____
(please provide name of diet and supplier)
☐ Other. Please specify:____
☐ I don't know
☐ Prefer not to answer

65. Did/do you provide any of the following feed supplements (extra on top of/separate from standard diet composition) to the chosen flock? *Select all that apply.*

☐ Poultry grit (e.g. sand)
☐ Insoluble fibre (e.g. whole oats, wheat, corn, alfalfa, barley etc.)
☐ Vitamins/minerals
☐ Other. Please specify:____
☐ No supplements provided
☐ I don't know
☐ Prefer not to answer

66. Do you have any animal by-products in your ration?

☐ Yes ☐ No ☐ I don't know ☐ Prefer not to answer

Watering 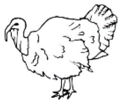

67. Please indicate the number of birds per drinker/cup in the barn____________________

68. Please specify the type of drinkers in the chosen barn:

☐ Closed watering system (i.e. nipple-type drinker)
☐ Open watering system (i.e. bell-type drinker, trough)
☐ Other. Please specify:____
☐ I don't know
☐ Prefer not to answer

69. What is the source of the water?

☐ Deep well ☐ Surface well (dugout)
☐ Pond water ☐ Town (municipal) water
☐ Other. Please specify:____
☐ I don't know
☐ Prefer not to answer

70. Did/do add any water sanitizing products to the water of the chosen flock? *Select all that apply*

☐ Chlorine ☐ Iodine
☐ Chlorine dioxide ☐ Hydrogen peroxide
☐ Ozone ☐ None
☐ Other. Please specify:____
☐ I don't know
☐ Prefer not to answer

71. Did/do add any water supplements to the water of the chosen flock? *Select all that apply*
☐ None
☐ Vitamins/minerals
☐ Acid
☐ Other. Please specify:____
☐ I don't know
☐ Prefer not to answer

72. Did you flush/clean/sanitize the watering lines before the placement of the chosen flock?

☐ Yes ☐ No ☐ I don't know ☐ Prefer not to answer

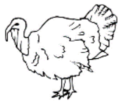

73. What do you look for when checking the water quality of the chosen flock? *Select all that apply*

☐ Rust
☐ Cloudiness
☐ Colour
☐ Odour
☐ Laboratory checks (CFUs, chemical content)
☐ Do not check water quality
☐ Other. Please specify:____
☐ I don't know
☐ Prefer not to answer

74. Please indicate the average daily water consumption of the chosen flock*:

_________________litres/day
_________________gallons/day
*Please answer with your preferred unit of measurment

4. Flock health – Chosen Flock 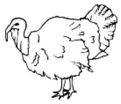

75. How did you prepare the barn before the birds arrived? *Select all that apply.*

☐ Dry cleaning
☐ Wet cleaning
☐ Disinfecting
☐ Heat treatment
☐ Downtime period
☐ Fumigation
☐ Other. Please specify:____
☐ I don’t know
☐ Prefer not to answer

76. Which of the following biosecurity practices do you apply? *Select all that apply.*

☐ Boots – washing/boot dips
☐ Vermin control (i.e. rodents, beetles, flies)
☐ Inspect birds from youngest to oldest
☐ Down-time after contact with other farms/species
☐ Hand washing before entry
☐ Single-use boot covers
☐ Shower in/out of barn
☐ Specific clothing for each barn
☐ I don’t know
☐ Prefer not to answer

77. Are poultry species other than turkeys (e.g. chickens) present on the farm site?

☐ Yes ☐ No ☐ I don’t know ☐ Prefer not to answer

78. Are any other animal species (e.g. cattle, sheep, goats, horses and/or pigs) other than poultry present on the farm site?

☐ Yes ☐ No ☐ I don’t know ☐ Prefer not to answer

79. How many times per day is the chosen flock inspected at their current age?

☐ Less than once ☐ Once ☐ Twice
☐ Three times ☐ Four times ☐ More than four times
☐ I don’t know ☐ Prefer not to answer

80. In one day, how many different people inspect the chosen flock?

☐ 1 ☐ 2 ☐ 3 ☐ 4 ☐ 5 ☐ More than 5
☐ I don’t know ☐ Prefer not to answer

81. On average, how long does a single inspection of the chosen flock take?

☐ Less than 15 min ☐ 15-30 min ☐ 30-45 min ☐ 45-60 min ☐ More than 60 min
☐ I don’t know ☐ Prefer not to answer

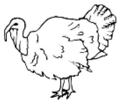

82. How do birds in the chosen flock react to you when you first enter the barn?

☐ No reaction ☐ Curious/Crowd around/Follow
☐ Flighty/Run away ☐ Other. Please specify:___________________________
☐ I don't know ☐ Prefer not to answer

83. What do you look for during inspections? *Select all that apply.*

☐ Dead birds
☐ Sick birds
☐ Quality of droppings
☐ Leg deformities/feet problems
☐ Head or neck injuries
☐ Breast injuries
☐ Back or tail injuries
☐ Wing injuries
☐ Feather damage/naked spots
☐ Varying body size
☐ Behaviour of the birds
☐ Vocalization of the birds
☐ Pile-up/smothering
☐ Distribution of birds in the barn
☐ Functioning equipment (e.g., feeders/waterers)
☐ Spilled feed
☐ Water leaks
☐ Water quality
☐ Litter conditions (e.g., wet litter, litter depth)
☐ Ventilation
☐ Temperature
☐ Lighting
☐ Condensation
☐ Ammonia levels
☐ Feed and water consumption
☐ Other. Please specify:____
☐ I don't know
☐ Prefer not to answer

84. How often do you pick up an individual bird during an inspection of the chosen flock?

☐ Never ☐ Sometimes ☐ About half of the time
☐ Most of the time ☐ Always
☐ I don't know ☐ Prefer not to answer

85. Were vaccines administered at the hatchery or on farm to the chosen flock? If yes, please indicate vaccine name or disease vaccinated against below.

☐ Yes ☐ No ☐ I don't know ☐ Prefer not to answer

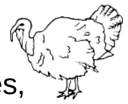

86. Were any medications administered to the chosen flock (i.e. medicated feed)? If yes, please complete table below.

☐ Yes ☐ No ☐ I don't know ☐ Prefer not to answer

Medication Name	Route<br>(i.e. feed, water, injection, spray etc.)	Purpose<br>(i.e. promote gut health)

87. Do you separate injured/sick birds from the flock to treat (i.e. hospital or recuperation pens)?

☐ Yes ☐ No ☐ I don't know ☐ Prefer not to answer

88. Were any of the following problems observed in the chosen flock? *Select all that apply*

☐ Turkey rhinotracheitis (avian pneumovirus)
☐ E. coli
☐ Reo virus
☐ Haemorrhagic enteritis
☐ Blackhead Disease
☐ Staphylococcus
☐ Cellulitis
☐ Mycoplasma
☐ Coccidiosis
☐ Salmonella
☐ Necrotic enteritis
☐ Worms, lice, mites
☐ Erysipelas
☐ Fowl cholera
☐ Bordetellosis
☐ None
☐ Other. Please specify:______________________________
☐ I don't know
☐ Prefer not to answer

89. Have you noticed any birds with poor vision, blindness, or cataracts in the chosen flock?

☐ Yes ☐ No ☐ I don't know ☐ Prefer not to answer

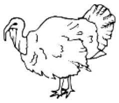

90. What is the total number of birds in this flock that have been culled since placement from the hatchery?

☐ I don't record culls and mortality separately (skip to number 89)
___________________ birds
___________________% cumulative culls

91. What is the total number of birds in this flock that have died since placement from the hatchery?

___________________ birds
___________________% cumulative mortality

92. Please select the 3 main reasons for culling birds in this flock:

☐ Pecking injuries
☐ Immobile/sickly
☐ Small body size (poor growth)
☐ Leg injuries (lameness, broken legs)
☐ Leg deformities (i.e., cowboy leg, hockey stick leg, splay leg, etc.)
☐ Pendulous crop
☐ Breast buttons or blisters
☐ Wing injuries
☐ Other. Please specify:____
☐ I don't know
☐ Prefer not to answer

93. Please select the 3 main causes of mortality in this flock:
☐ Dehydration – birds too small to reach drinker
☐ Smothering (i.e. pile-up or crowding of birds)
☐ Cannibalism (i.e. death due to excessive pecking by other birds)
☐ Disease/Illness. Please specify: ____
☐ Mechanical failure (i.e. heating/ventilation malfunction)
☐ Unknown cause of mortality
☐ Other. Please specify:____
☐ I don't know
☐ Prefer not to answer

5. Final comments

Please answer the remaining questions for your entire farm.

94. Please select the 3 most common causes of carcass condemnation or downgrading during processing on your farm:

☐ Respiratory conditions
☐ Sub-cutaneous conditions (Sub-cut conditions)
☐ Leg injuries (i.e. broken legs, footpad dermatitis)
☐ Leg deformities (i.e. cowboy leg, hockey stick leg, splay leg)
☐ Skin conditions (i.e. breast buttons or blisters)
☐ Fluid in abdomen
☐ Liver conditions
☐ Emaciation (birds too thin)
☐ Dark-coloured carcasses
☐ Ruptured tendons
☐ Other. Please specify:____
☐ I don't know
☐ Prefer not to answer

95. Please rate how much of an issue the following items are on your farm <u>in the summer</u>:

	No issue at all	A small issue	A big issue	I don't know	Prefer not to answer
Mortality	☐	☐	☐	☐	☐
Aggressive pecking	☐	☐	☐	☐	☐
Disease	☐	☐	☐	☐	☐
Leg injuries*	☐	☐	☐	☐	☐
Leg deformities**	☐	☐	☐	☐	☐
Breast injuries	☐	☐	☐	☐	☐
Varying body size	☐	☐	☐	☐	☐
Other. Please specify:	☐	☐	☐	☐	☐

*including lameness and foot pad dermatitis
**including cowboy leg, hockey stick leg, splay leg etc.

96. Please rate how much of an issue the following items are on your farm <u>in the winter</u>:

**Same as #95 ☐**	No issue at all	A small issue	A big issue	I don't know	Prefer not to answer
Mortality	☐	☐	☐	☐	☐
Aggressive pecking	☐	☐	☐	☐	☐
Disease	☐	☐	☐	☐	☐
Leg injuries*	☐	☐	☐	☐	☐
Leg deformities**	☐	☐	☐	☐	☐
Breast injuries	☐	☐	☐	☐	☐
Varying body size	☐	☐	☐	☐	☐
Other. Please specify:	☐	☐	☐	☐	☐

*including lameness and foot pad dermatitis
**including cowboy leg, hockey stick leg, splay leg etc.

97. Please rate how much of an issue the following items are <u>for the turkey production sector:</u>

**Same as #95** ☐	No issue at all	A small issue	A big issue	I don't know	Prefer not to answer
Mortality	☐	☐	☐	☐	☐
Aggressive pecking	☐	☐	☐	☐	☐
Disease	☐	☐	☐	☐	☐
Leg injuries*	☐	☐	☐	☐	☐
Leg deformities**	☐	☐	☐	☐	☐
Breast injuries	☐	☐	☐	☐	☐
Varying body size	☐	☐	☐	☐	☐
Other. Please specify:	☐	☐	☐	☐	☐

*including lameness, broken legs, foot pad dermatitis
**including cowboy leg, hockey stick leg, splay leg etc.

98. Would you be willing to participate in future research?

☐ Yes ☐ No ☐ I don't know

Final comments – *Please provide any feedback you wish to give regarding the questionnaire or the research project*

You have reached the end of the questionnaire. Please return the filled in <u>questionnaire</u> and turkey health scoring <u>recording sheet</u> back to the research team using the enclosed envelope. Thank you for your participation!

We appreciate your commitment to this research and look forward to working with you to continually improve the turkey production industry!
